# KDM5B promotes tumorigenesis of Ewing sarcoma via FBXW7/CCNE1 axis

**DOI:** 10.1038/s41419-022-04800-1

**Published:** 2022-04-15

**Authors:** Binbin Chen, Huimou Chen, Suying Lu, Xiaoqin Zhu, Yi Que, Yu Zhang, Junting Huang, Li Zhang, Yu Zhang, Feifei Sun, Juan Wang, Jia Zhu, Zijun Zhen, Yizhuo Zhang

**Affiliations:** 1 State Key Laboratory of Oncology in South China, Collaborative Innovation Center for Cancer Medicine, Sun Yat-sen University Cancer Center, Guangzhou, 510060 PR China; 2 Department of Clinical Nutrition, Sun Yat-sen University Cancer Center, Guangzhou, 510060 PR China; 3 Department of Pediatric Oncology, Sun Yat-sen University Cancer Center, Guangzhou, 510060 PR China; 4 Department of Pathology, Sun Yat-sen University Cancer Center, Guangzhou, 510060 PR China

**Keywords:** Paediatric cancer, Epigenetics, Prognostic markers

## Abstract

Ewing sarcoma (EwS) is an aggressive tumor that affects children and young adults. Patients with relapsed/refractory diseases have limited treatment options. Targeting the driver fusion oncoproteins of EwS remains a technical problem. Epigenetic mechanisms have been pointed out as key players and alternative therapeutic targets in EwS. Here, we reported that lysine demethylase 5B (KDM5B), a histone demethylase that specifically demethylates tri- and di-methylated H3 Lys-4 (H3K4), was upregulated in EwS and overexpressed KDM5B was correlated with poor outcomes of patients. KDM5B knockdown and KDM5B inhibitor AS-8351 suppressed EwS cell proliferation and induced cell cycle arrest. Bioinformatics analysis revealed that KDM5B mainly influenced the cell cycle pathways in EwS. In mechanistic studies, we found that overexpression of KDM5B resulted in increased CCNE1 protein level, but did not affect the mRNA level of *CCNE1*. KDM5B upregulation blocked the degradation pathway of CCNE1 by reducing the expression of FBXW7. KDM5B downregulated *FBXW7* gene by demethylation of H3K4me3 at promoter region. Moreover, AS-8351 could inhibit tumor growth in nude mice models, indicating the antitumor effect of targeting KDM5B in EwS. Our study uncovered that KDM5B in EwS attenuated *FBXW7* transcription and accumulated CCNE1 protein, leading to malignant proliferation of EwS. Epigenetic drug targeting KDM5B could be a potential treatment for EwS.

## Introduction

Ewing sarcoma (EwS), a highly aggressive bone and soft tissue tumor, mainly affects children and adolescents and has an incidence of about 1.5 per million per year [1]. EwS is characterized by high metastatic propensity and high recurrence rate after treatment [2]. Although multimodal therapies have improved survivals of patients with limited-stage diseases [3], those who have extensive-stage diseases or suffer disease progression still have extremely poor outcomes due to very limited treatment options [4, 5].

EwS is driven by characteristic chromosomal translocations which generate genomic rearrangements of the *EWS breakpoint region 1* (*EWSR1*) gene with members of ETS-family transcription factor genes, most commonly to *FLI1* [6]. The *EWS/ETS* fusion gene encodes aberrant transcription factors and post-transcriptional regulators, which causes a wide range of gene expression disorders, ultimately leading to the occurrence of malignancy. However, direct pharmacological targeting of the EWS/ETS fusion proteins has always been a daunting challenge, and such proteins are generally considered undruggable due to a lack of enzymatic activity. Therefore, uncovering other molecular mechanisms underlying EwS formation and progression is essential for identifying novel and effective therapeutic targets in EwS.

Many recent studies in the field of pediatric cancer have emphasized the importance of epigenetic mechanisms in the development of diseases [7], while epigenetic regulator, as a new and tractable therapeutic target in all types of cancer, has provoked a great interest [8]. Epigenetic mechanism has recently become a very important participant in the pathogenesis of EwS [9, 10], and a number of studies have identified that EWS/ETS fusion proteins involved in histone modification [2, 11, 12]. Jumonji-domain histone demethylases (JHDMs) are a large family of proteins that regulate the methylation status of lysine residues in histones [13, 14], and gradually become emerging therapeutic targets in recent years [15, 16]. Previous studies have demonstrated that JHDMs inhibitors manifested antitumor activity in multiple cancer types, including EwS [17, 18].

Lysine demethylase 5B (KDM5B), one member of the JHDMs family, is responsible for erasing tri‐ and dimethyl modifications of H3 lysine 4 (H3K4) [19]. The expression of KDM5B is limited in normal human adult tissues, mainly in testis and brain [20, 21]. Recently, cumulative studies have shown that KDM5B is overexpressed in a variety of human cancers, including breast cancer, gastric cancer, lung cancer, and prostate cancer, demonstrating its oncogenic function [22]. KDM5B has attracted extensive attention and is regarded as a promising drug target of cancer therapy [23]. However, the roles and mechanisms of KDM5B in EwS still have not been elucidated. In this study, we identified that KDM5B was highly expressed in EwS. KDM5B knockdown and KDM5B inhibitor AS-8351 could inhibit EwS cell proliferation and induce cell cycle arrest. Further investigation showed that KDM5B maintained the proliferative phenotype of EwS by regulating the FBXW7/CCNE1 axis. Moreover, AS-8351 could inhibit tumor growth in nude mice models, indicating the anti-tumor effect of targeting KDM5B in EwS. Our results provided new insights into the clinical treatment strategy for EwS by targeting this novel signaling pathway.

## Materials and methods

### Cell culture and compounds

Human EwS cell lines A673, RDES and SKNMC were cultured in RPMI 1640 with 10% fetal bovine serum (FBS); human mesenchymal stem cells (hMSC) and HEK-293T cells were cultured in DMEM with 10% FBS. All the above cell lines were purchased from American Type Culture Collection and were repeatedly verified to be mycoplasma-free. Cells were cultured at 37 °C with 5% CO_2_. AS-8351 was obtained from TargetMol (Shanghai, China). For in vitro studies, AS-8351 was dissolved in DMSO according to the manufacturer’s instructions; for in vivo (animal tumor) studies, AS-8351 was administered by oral gavage dissolved in saline containing 5% DMSO, 1% Tween80, and 30% PEG300.

### Tissue sample collection and ethics

A total of 46 archived formalin‐fixed, paraffin‐embedded EwS specimens, which were pathologically diagnosed at our center from 2002 to 2018, were collected for this study. Enrolled patients should receive systematic treatments and complete follow-ups until death or the end of the study. Endpoints of this study were overall survival (OS, defined as time interval between diagnosis and death from any cause) and event-free survival (EFS, defined as time interval between diagnosis and first disease progression or death from any cause). Patients without disease progression or those lived at the end of this study were treated as censored data. This study was approved by the Institutional Review Board and the Research Ethics Committee of Sun Yat-sen University Cancer Center. Informed consent was obtained from all eligible patients. All patients were treated under the principles of the Declaration of Helsinki.

### Bioinformatic analysis

EwS‐related differentially expressed genes were identified after differential analysis of the Gene Expression Omnibus (GEO) microarray database GSE17674 using the R package *limma* (http://www.bioconductor.org/packages/release/bioc/html/limma.html). Differentially expressed genes (DEGs) were identified using adjusted *P* value < 0.05 and | logFC| ≥ 1. GEO datasets of GSE17674, GSE12102, and GSE34620 were merged after selecting tumor samples from each dataset, and *ComBat* method (“sva” Biocoductor package) was used to correct the batch effects. Gene Ontology (GO) enrichment analysis, Kyoto Encyclopedia of Genes and Genomes (KEGG) pathway analysis, Spearman correlation analysis and Gene Set Enrichment Analysis (GSEA) are based on R language using the merged GEO dataset.

### RNA sequencing and data analysis

Cells were incubated with vehicle or AS-8351 for 48 h, after which total RNA was extracted using TRIzol reagents (Invitrogen, USA). Then, cDNA library building and RNA sequencing (RNA-seq) were performed via a commercially available service (service ID # F21FTSSCWLJ0737, BGI, Huada Gene, Wuhan, China). RNA-seq reads were aligned to the hg38 genome. DEGs were identified using |log2FC| ≥ 1 and FDR ≤ 0.001. The KEGG enrichment analysis and the Venn diagram for DEGs were performed by the BGI, using the Dr. TOM approach, an in-house customized data mining system of the BGI.

### Immunohistochemistry (IHC) analysis

The formalin-fixed paraffin-embedded tissues were sliced into 4 μM sections and then deparaffinized with xylene and rehydrated with graded ethanol. Endogenous peroxidase activity was blocked by incubation in 3% hydrogen peroxide for 10 min, then the slides were pressure cooked and boiled in sodium citrate buffer (pH 8.0) for antigen retrieval. Nonspecific antigens were blocked by incubation in 10% serum for 30 min at room temperature. Slides were incubated overnight at 4 °C with anti-KDM5B (1:200, Invitrogen, PA5-55535), anti-FBXW7 (1:200, Abcam, ab109617), anti-Ki67 (1:4000, ProteinTech, 27309-1-AP), and anti-CD99 (1:100, ProteinTech, 23079-1-AP).

All slides were examined by two independent pathologists (LZ and YZ). Five sights were selected in the high-power field (200×) randomly and observed with light microscope. KDM5B was mainly located in the nucleus, and its immunostaining was scored by the intensity of staining as 0 (negative) and 1 (positive). FBXW7 was distributed in the cytoplasm, and its immunostaining was scored by evaluating the staining intensity and percentage of positive cells. The intensity of FBXW7 staining was scored as 0 (none), 1 (weak), and 2 (marked). Percentage scores were assigned as follows: 1, 1–25%; 2, 26–50%; 3, 51–75%; and 4, 76–100%. The scores were multiplied to give a final score of 0 to 8 and the total expression of FBXW7 was determined as either low expression (score < 4) or high expression (score ≥ 4).

### Lentiviral infection

Small hairpin RNAs (shRNAs) against KDM5B (shKDM5B) or empty vectors (shNC) were packaged using second-generation lentiviral packaging systems. Briefly, 5 × 10^6^ HEK-293T cells were seeded in 10 cm dishes until the cell density reaches to 70–80%. ShRNA plasmids (10 μg) were co-transfected with pMD2.G (3 μg) and psPAX2 (7.5 μg) using 61.5 μL PEI transfection reagent (ProteinTech, China). The culture medium was collected after 48 h of transfection, then centrifuged and filtrated through 0.45 μm syringe filters before infection.

### Small interfering RNA (siRNA) and plasmids transient transfection

A673 and RDES cells were transiently transfected with 20 nM FBXW7 (siFBXW7) or a scrambled control (siNC) siRNA using Lipofectamine 3000 (Thermo Fisher Scientific, USA) following the manufacturer’s protocol. For plasmid transfection, 5 μg FLAG-tagged human CCNE1 overexpression plasmids (CCNE1-OE) or vector plasmids were transiently transfected in cells using Lipofectamine 3000. The cells were used for experiments after 24 h of transfection, or as otherwise indicated.

### RNA extraction and reverse transcription quantitative polymerase chain reaction (RT‐qPCR)

Total RNA was isolated from cells using the TRIzol reagent (Invitrogen, USA). Reverse transcription of cDNA was performed using the Fast All-in-One RT Kit with gDNA Remover (ESscience Biotech, Shanghai, China). Quantitative PCR was performed using Super SYBR Green qPCR Master Mix (ESscience Biotech, Shanghai, China) on PCR instrument (Bio-Rad, USA). Relative expression was calculated by the 2^−ΔΔCt^ formula after being normalized against *GAPDH* expression. The primer sequences used are provided in Supplementary Table S1.

### Western blot analysis

The protein concentration was measured using a bicinchoninic acid (BCA) assay (#23225; Pierce, USA) according to the manufacturer’s instructions. The primary antibodies used in this study are as followed: anti-KDM5B (1:500, Cell Signaling Technology, #15327), anti-H3K4me3 (1:1000, Cell Signaling Technology, #9751), anti-H3 (1:1000, Cell Signaling Technology, #4499), anti-CCNE1 (1:1000, Cell Signaling Technology, #20808), anti-CCNE2 (1:1000, ProteinTech, 11935-1-AP), anti-CDK2 (1:1000, ProteinTech, 10122-1-AP), anti-FLAG (1:1000, Cell Signaling Technology, #8146), anti-FBXW7 (1:1000, Abcam, ab109617), anti-FLI1 (1:1000, Abcam, ab133485), and β-actin (1:5000, Cell Signaling Technology, #4970). The secondary antibodies are goat anti‐rabbit immunoglobulin G (1:2000, Abcam, ab7090) and goat anti‐mouse immunoglobulin G (1:2000, Abcam, ab97040). The unprocessed western blot images were shown in Supplementary Fig. S2.

### Chromatin immunoprecipitation (ChIP) assays

ChIP assay was performed to analyze the enrichment of H3K4me3 binding at promoter regions of FBXW7 using a Magna ChIP Kit (Merck Millipore, USA) according to the manufacturer’s protocol. After reaching 70–80% confluence, cells were fixed by 1% formaldehyde at room temperature for 10 min to induce DNA-protein cross-linking, and the reaction was stopped by the addition of glycine. After washing, the cell lysates were treated by ultrasonication to shear the DNA into fragments of 200–1000 bp. Chromatin supernatants were incubated with anti-KDM5B (Cell Signaling Technology, #15327), anti-H3K4me3 (Cell Signaling Technology, #9751), anti‐RNA polymerase II (Millipore, #05‐623), or anti‐immunoglobulin G (IgG) (Sigma‐Aldrich, #I8765) overnight at 4 °C with rotation. Subsequently, Protein A/G magnetic beads were added to precipitate the antibody-target protein-DNA complexes, with the supernatant discarded. After washing, de‐cross‐linking and purification, enriched DNA fragments were subjected to qPCR analysis. IgG was used as the negative control. The fold change in the amount of the DNA fragments enriched by a specific antibody relative to the input sample was calculated by the following formula: %input = 100 × 2^[(Ct(input) − log(*x*%)/log2)-Ct(sample)], where *x*% represented the percentage of input sample in the total DNA. The primers used in the ChIP-qPCR assays are listed in Supplementary Table S1.

### Cell proliferation assays

Cells were seeded in 96-well plates at a concentration of 3000 cells per well in 200 μL of medium. After cultivation for the indicated time, the medium was removed and 100 μL diluted CCK-8 solutions (APExBIO, USA) were added. The plate was continued incubated at 37 °C for 2 h and the absorbance was measured at 450 nm. All experiments were performed in three independent trials.

### Colony formation assays

Cells were seeded in six-well plates at a concentration of 1000 cells per well. Cell culture medium was changed every three days. After 2 weeks of culture, the medium was removed and the colonies were fixed in 10% formalin solution for 2 h, with 0.1% crystal violet solution staining subsequently for another 10 min at room temperature. Digital images of the plates were taken for permanent record, with colonies counting by Image J software (version 1.8.0).

### Apoptosis assays

For cell apoptosis assay, Annexin V-Alexa Fluor 647/7-AAD Apoptosis Detection Kit (4A Biotech, China) was used according to the manufacturer’s instructions. Briefly, cells were collected, washed, and resuspended in binding buffer, stained with Annexin V-Alexa Fluor 647 and 7-AAD sequentially, and immediately analyzed on the CytoFLEX flow cytometer (Beckman Coulter, USA).

### Cell cycle analysis

Cells were induced cell cycle synchronized by serum starvation for 24 h, and then replaced by medium with 10% serum for 48 h culture. After incubation, cells were harvested and fixed in 75% cold ethanol at 4 °C overnight prepared. The fixed cells were centrifuged at 1000 *g* for 5 min and washed twice using cold phosphate-buffered saline. The treated cells were stained with propidium iodide (0.05 mg/mL) and RNase A (2.0 mg/mL), and incubated for 30 min at 37 °C in the dark. Then, the stained cells were placed on the CytoFLEX flow cytometer (Beckman Coulter, USA) for cell cycle analysis. The cell percentages in G0/G1, S, and G2/M phases were further analyzed with the FlowJo software (version 10.2).

### In vitro assays of drug sensitivity

Cells were plated at a concentration of 5000 cells per well in 100 μL of medium and allowed to attach overnight. The medium was then aspirated away and replenished by medium containing desired concentrations of AS-8351 or vehicle control. After incubation for the indicated time, the medium was removed and 100 μL diluted CCK8 solutions (APExBIO, USA) were added for another incubation of 2 h at 37 °C. Absorbance was measured at 450 nm. Cell viability was calculated by the following formula: cell viability rate = [(As − Ab)/(Ac − Ab)] × 100, where As represented the absorbance of experimental wells, Ab represented the absorbance of blank wells, and Ac represented the absorbance of vehicle control wells. The half-maximal inhibitory concentration (IC50) values were determined via a nonlinear regression plot performed by the GraphPad Prism software (version 8.2.1) based on the cell viability rate.

### Tumor xenograft studies

In vivo studies were performed after the approval of the Institutional Animal Care and Use Committee of Sun Yat-sen University Cancer Center. A total of twelve 4- to 6-week-old female nude mice were injected subcutaneously into the right flank with 5 × 10^6^ A673 cells. When the tumors reached to 50–100 mm^3^, mice were randomly divided into vehicle control group and AS-8351 treatment group. The investigators were not blinded to the group allocation. AS-8351 was administered by oral gavage at a dose of 60 mg/kg/d for 10 days. Tumors were measured using digital calipers every 2 days. Tumor volumes were calculated by the following formula: volume = 0.5 × length × width^2^. The body weights of mice were monitored throughout the course of the study. When tumor volumes reached the institutional limit of 2000 mm^3^, mice were sacrificed and transplanted tumors were extracted for further analysis.

### Statistical analysis

Statistical analysis was performed using GraphPad Prism software (version 8.2.1) or SPSS statistics software (version 25). Continuous variables were expressed as mean ± standard deviation, and differences were compared using Student’s *t* test or one-way analysis of variance. The differences of categorical variables were compared using the χ^2^ test or Fisher’s exact test. Survival rates were calculated by Kaplan-Meier method and the comparisons were performed using Log-rank test. All tests were two-sided, and *P* < 0.05 was considered to be statistically significant.

## Results

### KDM5B was upregulated in EwS tissues and was associated with a poor prognosis

**Fig. 1:**
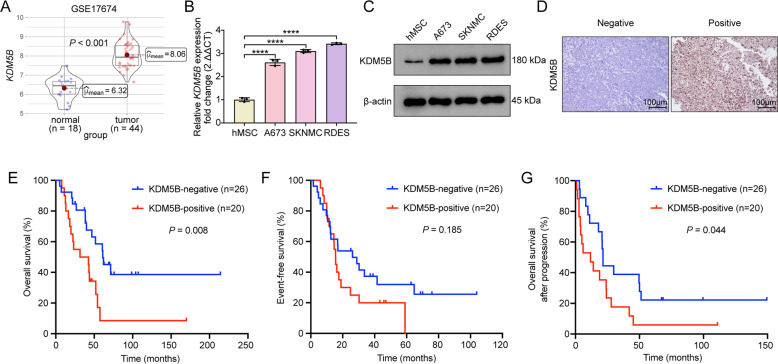
KDM5B is upregulated in Ewing sarcoma and is associated with poor prognosis. **A** Box plot of *KDM5B* mRNA expression in GSE17674 between Ewing sarcoma samples and normal tissues. **B** KDM5B expression in Ewing sarcoma cell lines A673, SKNMC and RDES versus in normal cell line hMSC assessed by RT‐qPCR and **C** western blot analysis. **D** Representative immunohistochemistry images of KDM5B protein expression in Ewing sarcoma samples. Scale bars: 100 μm. **E**–**G** Kaplan-Meier analysis of correlation of KDM5B protein expression with overall survival (OS), event-free survival (EFS) and OS after progression in 46 Ewing sarcoma patients at our center. Error bars in **B** indicate standard deviation of three biological independent experiments. *P* values in **A** are determined by two‐tailed unpaired Student’s *t* test. *P* values in **E**, **F**, **G** are determined by log-rank test. *Above the bars indicates significant difference; ****P < 0.0001.

To investigate the role of KDM5B in EwS, we investigated *KDM5B* mRNA expression levels in GSE17674 dataset containing 44 tumor tissues from EwS patients and 14 normal tissues from skeletal muscle. The volcano map of DEGs between EwS and normal tissues was shown in Supplementary Fig. S1A. By extracting the mRNA expression data of GSE17674, we confirmed that the expression of *KDM5B* was significantly elevated in EwS tissues than in normal tissues (Fig. 1A). Then, RT-qPCR and western blot analysis were used to determine the expression level of KDM5B in the human EwS-derived cell lines A673, SKNMC and RDES as well as hMSC which was proposed as a plausible cell of origin for EwS. The results revealed that the mRNA expression level of *KDM5B* was notably elevated in EwS-derived cell lines (Fig. 1B), and KDM5B protein expression in EwS cell lines was consistent with the RT-qPCR result (Fig. 1C). Furthermore, we explored the relationship between KDM5B expression and patient survival at our center. Detailed characteristics of 46 EwS patients were listed in Supplementary Table S2. Typical images of IHC analysis showing negative and positive KDM5B expression were presented in Fig. 1D. The clinical baseline characteristics of the two groups were balanced (Supplementary Table S3). Survival analysis indicated that positive KDM5B expression (*n* = 20) was correlated with poor OS (*P* = 0.008; Fig. 1E). Although EFS presented no statistically significant difference between the two groups (*P* = 0.185; Fig. 1F), the OS after progression was still better in the group of negative KDM5B expression (*n* = 26; *P* = 0.004; Fig. 1G). Collectively, these data suggested the potential clinical significance of KDM5B in EwS.

### KDM5B depletion impaired tumorigenesis of EwS cells in vitro

**Fig. 2:**
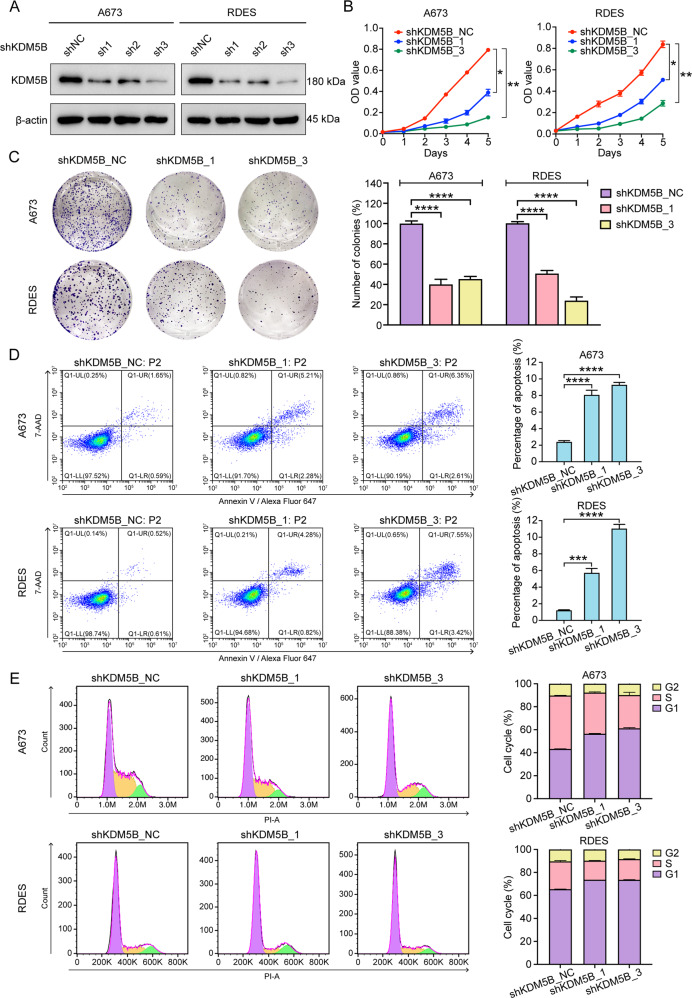
Knockdown of KDM5B inhibits the function of Ewing sarcoma cells. **A** Western blot analysis of KDM5B expression in A673 and RDES cells after shKDM5B_NC, shKDM5B_1, shKDM5B_2, and shKDM5B_3 treatment. **B** Growth curves of A673 and RDES cells after shKDM5B treatment. **C** Left panel: Representative images of colony formation assays of A673 and RDES cells after shKDM5B treatment. Right panel: Histogram of three biological independent experiments of colony formation assays. **D** Left panel: Representative images of cell apoptosis analysis of A673 and RDES cells after shKDM5B treatment using flow cytometry. Right panel: Histogram of three biological independent experiments of cell apoptosis analysis. **E** Left panel: Representative images of cell cycle analysis of A673 and RDES cells after shKDM5B treatment using flow cytometry. Right panel: Histogram of three biological independent experiments of cell cycle analysis. Error bars in **B**, **C**, **D**, **E** indicate standard deviation of three biological independent experiments. *P* values in **B** are determined by two‐way ANOVA test. *P*‐values in **C**, **D** are determined by two‐tailed unpaired Student’s *t* test. *Above the bars indicates significant difference; *P < 0.05, **P < 0.01, ***P < 0.001, ****P < 0.0001.

To investigate the potential role of KDM5B in EwS cells, we constructed KDM5B stable knockdown EwS cell lines A673 and RDES using three independent shRNAs. Western blot analysis confirmed the successful depletion of KDM5B expression in A673 and RDES cells by the three shRNAs, among which shKDM5B_1 and shKDM5B_3 displayed the better efficiencies (Fig. 2A) and were therefore used in subsequent experiments. As shown, KDM5B knockdown dramatically decreased the growth (Fig. 2B) and colony-formation (Fig. 2C) abilities of A673 and RDES cells. Moreover, KDM5B depletion promoted A673 and RDES cells apoptosis (Fig. 2D) and induced G1/S-phase cell cycle arrest (Fig. 2E). Taken together, these results indicated that KDM5B knockdown impaired EwS tumorigenesis in vitro.

### KDM5B inhibitor AS-8351 suppressed EwS cells proliferation

**Fig. 3:**
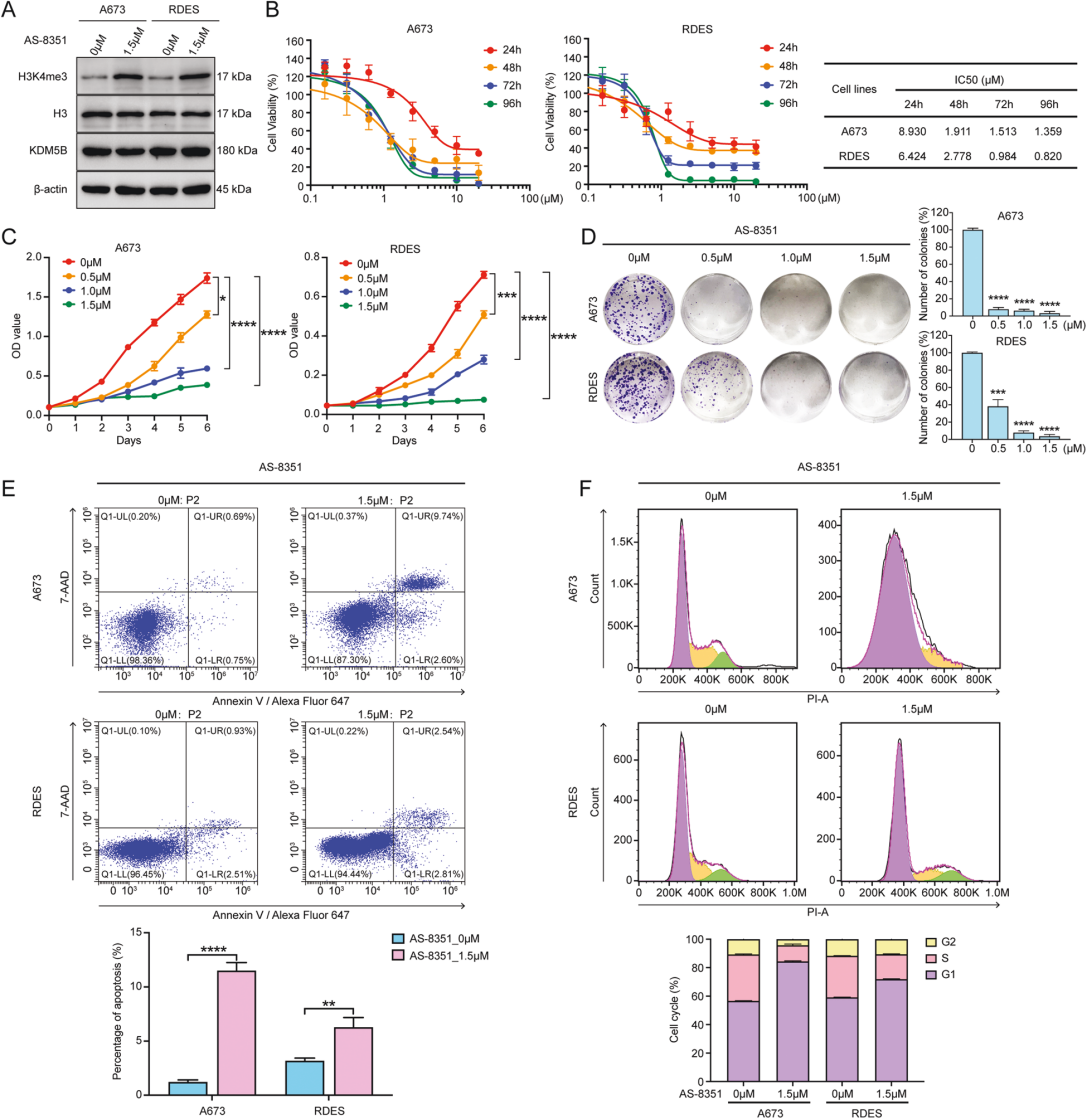
KDM5B inhibitor AS-8351 suppresses the function of Ewing sarcoma cells. **A** Western blot analysis of KDM5B and H3K4me3 expression in A673 and RDES cells after AS-8351 treatment. **B** Cell viability assay of AS-8351 against A673 and RDES cell lines. Statistical chart showing the IC50 values of AS-8351 against A673 and RDES cell lines at different time points. **C** Growth curves of A673 and RDES cells after AS-8351 treatment. **D** Left panel: Representative images of colony formation assays of A673 and RDES cells after AS-8351 treatment. Right panel: Histogram of three biological independent experiments of colony formation assays. **E** Up panel: Representative images of cell apoptosis analysis of A673 and RDES cells after AS-8351 treatment using flow cytometry. Down panel: Histogram of three biological independent experiments of cell apoptosis analysis. **F** Up panel: Representative images of cell cycle analysis of A673 and RDES cells after AS-8351 treatment using flow cytometry. Down panel: Histogram of three biological independent experiments of cell cycle analysis. Error bars in **B**, **C**, **D**, **E**, **F** indicate standard deviation of three biological independent experiments. *P* values in **C** are determined by two‐way ANOVA test. *P* values in **D**, **E** are determined by two‐tailed unpaired Student’s *t* test. *Above the bars indicates significant difference; *P < 0.05, **P < 0.01, ***P < 0.001, ****P < 0.0001.

AS-8351 is a new small molecule compound which is reported to inhibit KDM5B by competing with α-ketoglutarate for chelating Fe(II) [24]. In order to determine whether AS-8351 affected the growth of EwS cells, we examined its effect in an in vitro drug sensitivity assay. First of all, we verified that AS-8351 restrained the function of KDM5B, manifesting as an increase in its catalytic substrate H3K4me3 without reducing its own protein expression (Fig. 3A). Next, the cell viability assay of AS-8351 against A673 and RDES was tested. The results indicated that the longer time of drug exposure, the more obvious effect on the reduction of cell activity, with IC50 values of 1.911 μM in A673 and 2.778 μM in RDES after 48 h (Fig. 3B). Similarly, the cell proliferation assay also revealed that higher concentration of AS-8351 resulted in stronger inhibition of cell growth (Fig. 3C). Then, to investigate whether low cell density conditions would cause even greater drug sensitivity, clonogenic assay cultured with vehicle or different concentrations of AS-8351 was adopted. The results showed that AS-8351 inhibited EwS cell colony growth in a low micromolar range (Fig. 3D). Furthermore, we observed that AS-8351 promoted tumor cell apoptosis (Fig. 3E) and increased the proportion of G1/S phase‐arrested cells (Fig. 3F), which could be the reason for the inhibition of cell proliferation.

### KDM5B promoted the protein expression of CCNE1

**Fig. 4:**
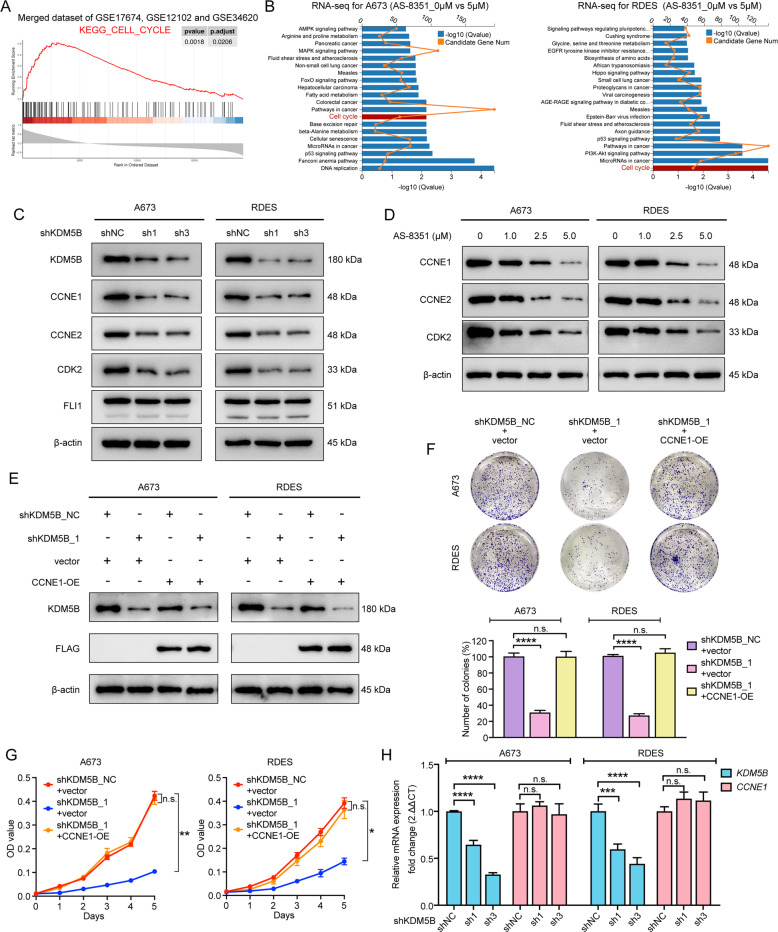
KDM5B activates cell cycle pathway of Ewing sarcoma cells and promotes protein expression of CCNE1. **A** Gene Set Enrichment Analysis (GSEA) showing activated cell cycle pathway by KDM5B in a merged dataset of GSE17674, GSE12102 and GSE34620. **B** KEGG enrichment analysis for RNA sequencing in A673 and RDES cells after vehicle or AS-8351 treatment. **C** Western blot analysis showing the expression of the key molecules of G1/S phase transition, CCNE1, CCNE2 and CDK2, and FLI1 in A673 and RDES cells after shKDM5B treatment. **D** Western blot analysis showing the expression of CCNE1, CCNE2 and CDK2, in A673 and RDES cells after AS-8351 treatment. **E** Western blot analysis of the protein expression in shKDM5B Ewing sarcoma cells treated with or without CCNE1-overexpression. **F** Up panel: Representative images of colony formation assays in shKDM5B Ewing sarcoma cells treated with or without CCNE1-overexpression. Down panel: Histogram of three biological independent experiments of colony formation assays. **G** Growth curves of shKDM5B Ewing sarcoma cells A673 and RDES treated with or without CCNE1-overexpression. **H** Relative mRNA expression of *CCNE1* in shKDM5B Ewing sarcoma cells. Error bars in **F**, **G**, **H** indicate standard deviation of three biological independent experiments. *P* values in **F**, **H** are determined by two‐tailed unpaired Student’s *t* test. *P* values in **G** are determined by two‐way ANOVA test. *Above the bars indicates significant difference; *P < 0.05, **P < 0.01, ***P < 0.001, ****P < 0.0001; n.s. represents no significance.

In order to investigate the mechanism underlying KDM5B in promoting cell proliferation, we performed public database analysis and RNA-seq analysis. For database analysis, we used a merged dataset of GSE17674, GSE12102 and GSE34620 to calculate the correlation coefficients between all genes and KDM5B by Spearman correlation analysis. GO analysis and KEGG analysis were performed for the genes with the top 1000 correlation coefficients. The result showed that the genes correlated to KDM5B were highly enriched in pathways associated with cell cycle control (Supplementary Fig. S1B and C). GSEA analysis revealed that KDM5B activated the cell cycle pathway (Fig. 4A and Supplementary Fig. S1D). Similarly, KEGG pathway analysis of our RNA-seq data also showed that DEGs between the vehicle and the AS-8351 treatment groups were enriched in the cell cycle pathway (Fig. 4B). Combined with our previous results that KDM5B inhibition induced G1/S-phase cell cycle arrest, we decided to determine the expression levels of key regulatory proteins of G1/S transition to further uncover the mechanism underlying KDM5B-mediated cell cycle arrest. Western blot analysis demonstrated that KDM5B knockdown by shRNAs or inhibition by AS-8351 reduced the expression of CCNE1, CCNE2 and CDK2, the checkpoint regulators required for the progression of the G1/S phase (Fig. 4C, D). Since EWS-FLI1 fusion protein is crucial for the development of EwS, we further explored whether KDM5B inhibition affected the expression of EWS-FLI1. The result showed that KDM5B knockdown did not alter the protein expression of EWS-FLI1 (Fig. 4C).

Numerous previous studies have reported that CCNE1, also called cyclin E or cyclin E1, is upregulated in several human cancers and functions as a well-known oncogene [25–29]. In addition, one study found that transgenic mice overexpressing human cyclin E could develop mammary carcinoma [30], suggesting that CCNE1 is crucial for oncogenic transformation. Therefore, CCNE1 was selected for downstream research in our study. To determine whether KDM5B promoted cell proliferation depending on CCNE1, we performed functional rescue assays by transiently overexpressing CCNE1 plasmid with FLAG tag (CCNE1-OE) and vector plasmid in KDM5B knockdown cells (Fig. 4E) and observed the change of their proliferation ability. The results revealed that, compared with the vector group, the CCNE1-OE group could significantly reverse the proliferation suppression causing by KDM5B depletion (Fig. 4F and G). Taken together, we believed that CCNE1 was a functional target of KDM5B. Since KDM5B is responsible for regulating the histone modification, playing a role in inhibiting the transcription of downstream genes, we further explored the mRNA expression level of *CCNE1* when knocking down KDM5B. The results showed that KDM5B depletion did not affect *CCNE1* expression at the transcriptional level (Fig. 4H), which prompted us that CCNE1 was not the direct target of KDM5B through the transcription mechanism.

### KDM5B inhibited the transcription of FBXW7

Above results suggested that it was at the post-transcriptional level that KDM5B up-regulated CCNE1 expression, not at the transcriptional level. It has been extensively reported that the most common post-transcriptional regulation of CCNE1 is degradation by ubiquitination, and the notable ubiquitin ligase that specifically recognizes CCNE1 as a substrate is FBXW7 [31–33]. As an important tumor suppressor gene, *FBXW7* has been proved to be mutated or epigenetically silenced in a variety of human cancers [34, 35]. On this basis, we assumed that FBXW7 might be the mediator between KDM5B and CCNE1.

**Fig. 5:**
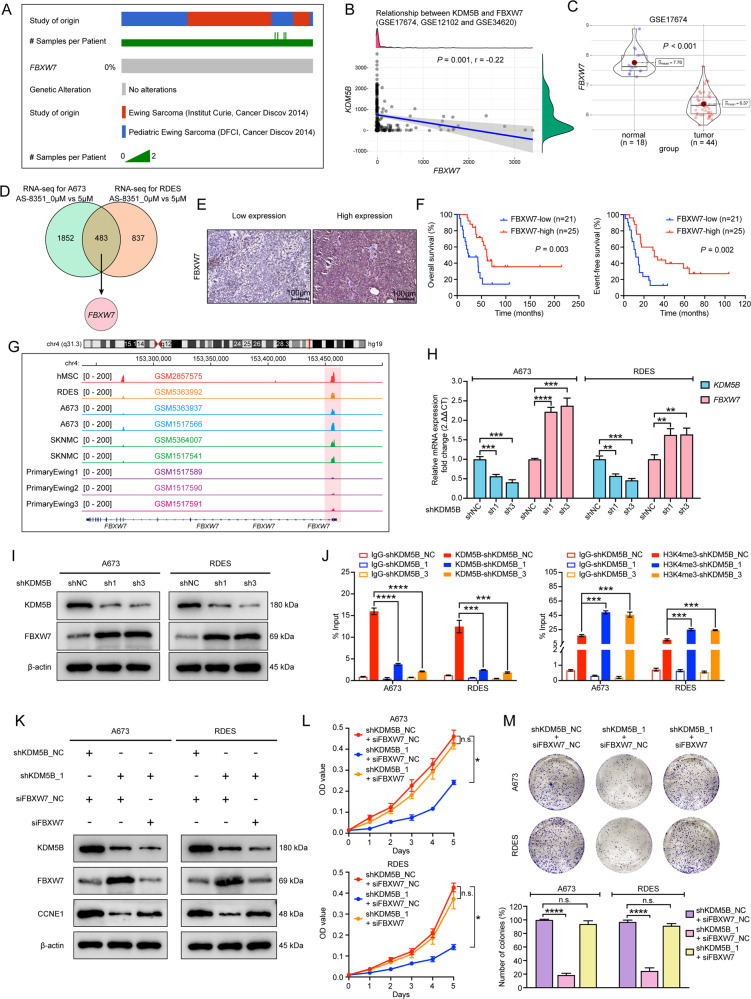
KDM5B transcriptionally down-regulates FBXW7 by reducing H3K4me3 level at the promoter region. **A** Oncoprint scheme showing alterations of *FBXW7* in Ewing sarcoma samples in cBioPortal online website (Pediatric Ewing Sarcoma, DFCI, Cancer Discov 2014 and Ewing Sarcoma, Institut Curie, Cancer Discov 2014). **B** Correlation diagram of *KDM5B* and *FBXW7* by spearman correlation analysis in a merged dataset of GSE17674, GSE12102 and GSE34620. **C** Box plot of *FBXW7* mRNA expression in GSE17674 between Ewing sarcoma samples and normal tissues. **D** Venn diagram from RNA sequencing data showing intersectional up-regulation of *FBXW7* in A673 and RDES cell lines after AS-8351 treatment. **E** Representative immunohistochemistry images of FBXW7 protein expression in Ewing sarcoma samples. Scale bars: 100 μm. **F** Kaplan-Meier analysis of correlation of FBXW7 protein expression with overall survival (left panel) and event-free survival (right panel) in 46 Ewing sarcoma patients. **G** Schematic diagram from GEO datasets showing H3K4me3 peak at the promoter region of *FBXW7* in Ewing sarcoma cells (RDES, A673 and SKNMC), primary Ewing sarcoma tissues and normal tissue (hMSC) using IGV software (version 2.11.9). **H** Relative mRNA expression of *FBXW7* in A673 and RDES cell lines treated with shKDM5B. **I** Western blot analysis of FBXW7 expression in A673 and RDES cells after shKDM5B treatment. **J** ChIP-qPCR assays showing KDM5B enrichment (left panel) and H3K4me3 enrichment (right panel) at *FBXW7* promoter in A673 and RDES cells after shKDM5B treatment. **K** Western blot analysis of the protein expression in shKDM5B Ewing sarcoma cells treated with or without siFBXW7. **L** Growth curves of shKDM5B Ewing sarcoma cells treated with or without siFBXW7. **M** Up panel: Representative images of colony formation assays in shKDM5B Ewing sarcoma cells treated with or without siFBXW7. Down panel: Histogram of three biological independent experiments of colony formation assays. Error bars in **H**, **J**, **L**, **M** indicate standard deviation of three biological independent experiments. *P* values in **F** are determined by log-rank test. *P* values in **H**, **J**, **M** are determined by two‐tailed unpaired Student’s *t* test. *P* values in **L** are determined by two‐way ANOVA test. *Above the bars indicates significant difference; *P < 0.05, **P < 0.01, ***P < 0.001, ****P < 0.0001; n.s. represents no significance.

Given the fact that *FBXW7* is prone to mutate in human cancer, we first determine whether *FBXW7* is mutated in EwS. We analyzed the landscape of *FBXW7* mutation in EwS on the cBioPortal online website (http://www.cbioportal.org) and found no mutation locus in the *FBXW7* gene in EwS patients (Fig. 5A). Next, we investigated if FBXW7 had any relevance to KDM5B. We analyzed a merged GEO dataset of GSE17674, GSE12102 and GSE34620 and found a significantly negative correlation between *KDM5B* and *FBXW7* mRNA expression (Fig. 5B). In GSE17674, *FBXW7* expression was down-regulated in EwS tissues than in normal tissues (Fig. 5C), which was just the opposite to the expression of *KDM5B*. Venn diagram from our RNA-seq data showed that *FBXW7* was one of the intersectional up-regulated DEGs when KDM5B was inhibited (Fig. 5D). Then, we explored whether FBXW7 expression influenced EwS patient survival at our center. Typical images of IHC analysis showing low and high expression of FBXW7 were presented in Fig. 5E. The clinical baseline characteristics of the two groups were balanced except for the chemotherapy courses (Supplementary Table S4). The Kaplan-Meier survival curves showed that low expression of FBXW7 was correlated with poor OS and EFS (*P* = 0.003 and *P* = 0.002, respectively; Fig. 5F) of 46 EwS patients at our cohort. Furthermore, published ChIP-seq data of EwS cells and primary EwS tissues manifested an H3K4me3 binding peak at the promoter area of the *FBXW7* gene, and the intensity of their H3K4me3 binding signals was lower than that of hMSC’s (Fig. 5G). The RT-qPCR and western blot analysis revealed that knockdown of KDM5B led to upregulation of FBXW7 (Fig. 5H and I). The above results intensively implied that FBXW7 might be the direct downstream target of KDM5B. To test this hypothesis, we detected the KDM5B and H3K4me3 signals at the promoter area of the *FBXW7* gene in cells knocking down of KDM5B. ChIP-qPCR showed that KDM5B silencing weakened KDM5B binding signal and enhanced H3K4me3 binding signal at the promoter area of the *FBXW7* gene (Fig. 5J). These data indicated that KDM5B inhibited the transcription of *FBXW7* by erasing tri-methylation modification of H3K4 at the promoter area.

### KDM5B promoted EwS cells tumorigenesis by regulating FBXW7/CCNE1 axis

Since FBXW7 is an ubiquitin ligase that targets CCNE1 for degradation, we examined whether KDM5B decreased CCNE1 degradation by suppressing FBXW7 expression. We transfected scrambled siRNA (siNC) or siRNA targeting FBXW7 (siFBXW7) in KDM5B knockdown cells. Western blot analysis revealed that FBXW7 depletion in KDM5B knockdown A673 and RDES cells up-regulated CCNE1 expression (Fig. 5K), supporting that KDM5B regulated CCNE1 by modulating FBXW7. Functional assays showed that FBXW7 depletion improved the growth (Fig. 5L) and colony-formation (Fig. 5M) abilities of KDM5B knockdown EwS cells. Collectively, these data suggested that KDM5B promoted EwS cells malignant proliferation through FBXW7/CCNE1 axis.

### KDM5B inhibitor AS-8351 impaired tumorigenesis of EwS cells in vivo

**Fig. 6:**
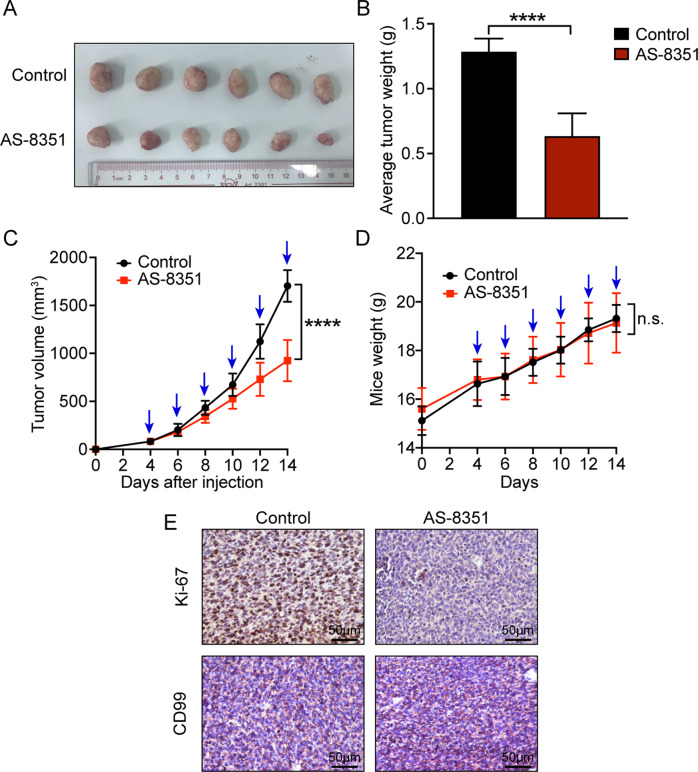
KDM5B inhibitor AS-8351 attenuates tumor growth of Ewing sarcoma cells in vivo. **A** Xenograft tumors extracted from mice models. **B** Box plot of xenograft tumor weight between the control and AS-8351 treatment groups. **C** Tumor growth curves of the xenograft tumors. Blue arrows indicate that AS-8351 is being used. **D** Mice weight curves during the experiment. Blue arrows indicate that AS-8351 is being used. **E** Representative immunohistochemistry images of Ki-67 and CD99 protein expression in xenografts at the end of the experiment. Ki-67 is used to assess cell proliferation. CD99, typically expressed on the cell membrane of Ewing sarcoma, is used to identify cancer cells from the stroma. Scale bars: 50 μm. Error bars in **B**, **C**, **D** indicate standard deviation. *P* values in **B** are determined by two‐tailed unpaired Student’s *t* test. *P* values in **C** and **D** are determined by two‐way ANOVA test. *Above the bars indicates significant difference; ****P < 0.0001; n.s. represents no significance.

Having verified the oncogenic role of KDM5B, we next explored the clinical significance of targeting KDM5B using AS-8351 in EwS by the in vivo xenograft growth model. As shown, mice treated with AS-8351 had significantly smaller tumors and slower tumor growth rates than those in the control group (Fig. 6A–C). Moreover, the mice body weights were comparable between the two groups during the experiment (Fig. 6D), indicating few toxicities and side effects of AS-8351. IHC staining also showed that tumors treated by AS-8351 had fewer Ki67-positive cells, where the tumor areas were expressed with high CD99 (Fig. 6E). Taken together, AS-8351 could inhibit tumor growth of EwS cells in vivo.

## Discussion

Epigenetic regulation has recently been confirmed playing a critical role in human cancers [36], particularly in pediatric cancers [7] which are reported to have few genetic mutations [37]. Ewing sarcoma is driven by *EWS/ETS* fusion gene which has been found to affect several epigenetic alterations, including histone modifications [2]. In the present study, we found that histone demethylase KDM5B was overexpressed in EwS and was correlated with poor outcomes of EwS patients. KDM5B knockdown by shRNAs or inhibition by AS-8351 reduced EwS cell proliferation and induced cell apoptosis and cell cycle arrest. Further investigations showed that KDM5B accumulated CCNE1 in tumor cells by decreasing the expression of FBXW7, which resulted in the acceleration of G1/S cell cycle phase transition and finally promoted malignant proliferation. Moreover, we found that AS-8351 inhibited the growth of EwS tumor xenografts without causing side effects in the mice, suggesting that epigenetic drug targeting KDM5B could be an effective anti-tumor agent for EwS treatment.

Previous studies have reported that KDM5B is essential for mammalian embryonic development in physiological conditions [21, 38, 39]. A study showed that newborn mice developed from KDM5B knockout embryos were lethal, mainly due to developmental defects in the respiratory system and nervous system [40]. KDM5B is involved in a range of human physiological processes as well, including genome stability and stem cell differentiation [19]. However, aberrant KDM5B expression can lead to tumorigenesis. Genome sequencing of human tumors unveiled a common mutation, amplification and overexpression of KDM5B in many cancer types [41]. In cancer cells, KDM5B regulates the expression of oncogenes and tumor suppressors by modulating H3K4 methylation levels, but the functions and mechanisms of KDM5B are not clear in EwS. In the present study, we discovered that KDM5B was highly expressed in EwS and associated with poor outcomes of patients. Knockdown of KDM5B significantly suppressed the cell proliferation of EwS. These suggested that KDM5B acted as an oncogene in EwS, which was similar to a previous research [42].

The physiological cell cycle relies on the orderly activation and degradation of cyclins and cyclin-dependent kinases in each phase. Among them, cyclins play a major role in regulating cycle phase transitions. Abnormal expression of cyclins could lead the cell cycle out of control, causing a series of terrible events such as chromosomal instability and abnormal mitosis, which ultimately results in the occurrence of tumors [43]. CCNE1, a member of cyclins, with the highest activity during G1/S phase transition, has been reported overexpressed in a variety of tumors [25–29], and has a strong carcinogenic effect in mice [30]. Our study found that KDM5B promoted the G1/S phase transition of EwS cells, which intrigued us to explore the relationship between KDM5B and CCNE1. Further experiments revealed indeed that KDM5B stimulated EwS cell growth by upregulating CCNE1.

It has been known that H3K4me3 at the promoter area is a marker of transcriptional activation [44]. Thus, demethylation of H3K4 is associated with transcriptional suppression, whereby KDM5B inhibits the transcription of downstream target genes. However, in the present study, we found that KDM5B increased the protein expression of CCNE1 without affecting its mRNA expression in EwS. On this basis, we believed that there should be an intermediate molecule between KDM5B and CCNE1. A previous research comprehensively demonstrated the current known mechanisms of CCNE1 upregulation including gene amplification, transcription up-regulation, and degradation interruption [33]. Combined with our results, we considered that KDM5B upregulated CCNE1 through blocking the protein degradation. A series of studies have shown that the most common degradation pathway of CCNE1 is ubiquitination, and the notable ubiquitin E3 ligase that specifically binds to CCNE1 is FBXW7 [31–33]. It has been reported that FBXW7 is usually low expressed in many human cancers and is well recognized as a tumor suppressor gene [34, 35]. Therefore, we speculated that FBXW7 might be the intermediate molecule between KDM5B and CCNE1. Further investigations proved our speculation that KDM5B inhibited the transcription of *FBXW7* by demethylation of H3K4 at the promoter area, leading to CCNE1 accumulation in cells, thereby promoting the cell cycle process and proliferation of tumors.

AS-8351, a new small molecule compound targeting KDM5B, has only been reported to inhibit tumor growth in breast cancer [45]. In this study, we verified the antitumor effect of AS-8351 in EwS in vivo and in vitro, and discovered that the toxicity of AS-8351 was tolerable to nude mice models. These results provided evidences for the clinical transformation of targeting KDM5B in EwS. However, further studies are needed before the application to clinical treatment.

**Fig. 7:**
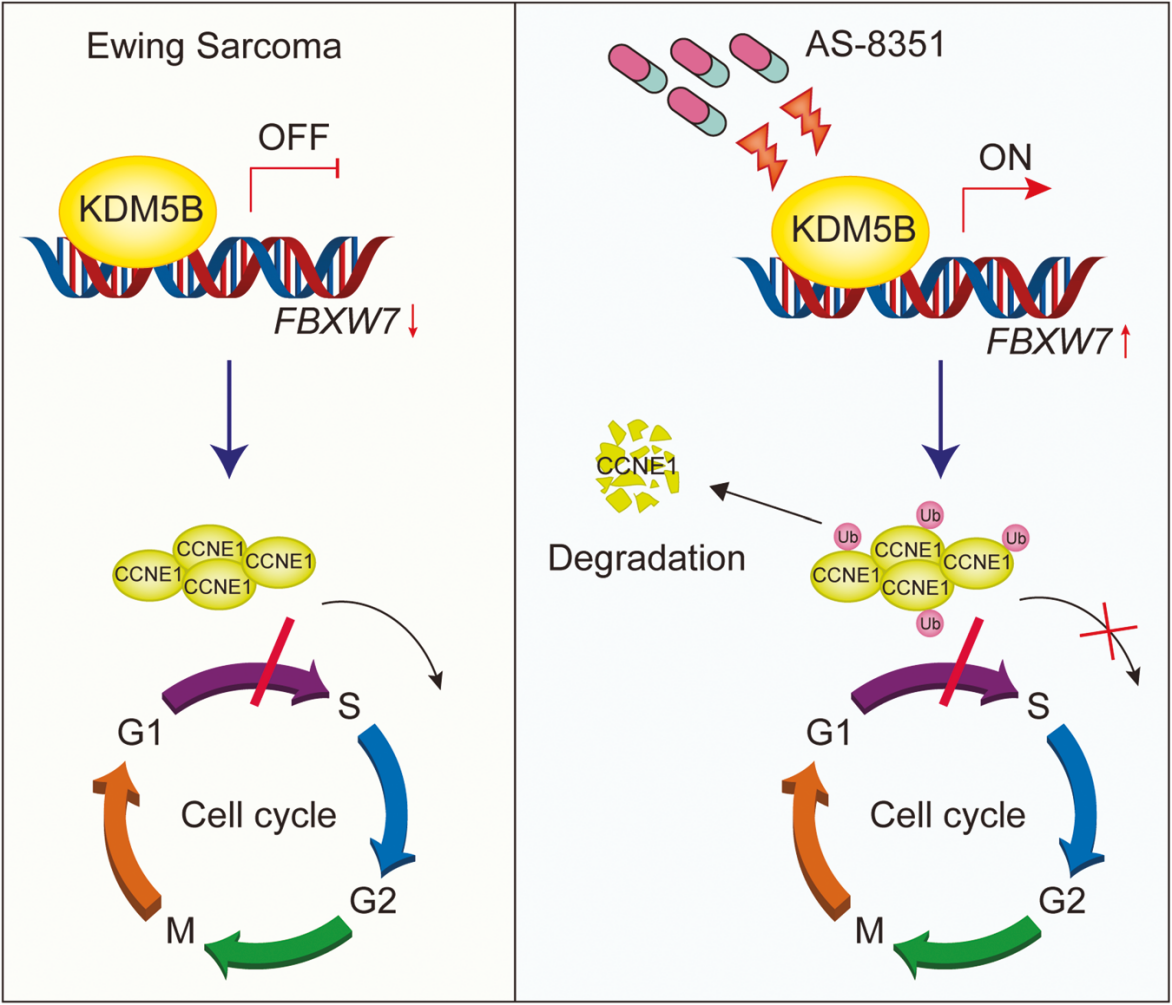
Schematic diagram of KDM5B-mediated regulation for Ewing sarcoma proliferation by the FBXW7/CCNE1 axis. (Left) In Ewing sarcoma cells, KDM5B overexpression inhibits *FBXW7* transcription by demethylation of H3K4me3 at the promoter region, blocking the degradation of CCNE1 and accumulating CCNE1 protein, leading to acceleration of cell cycle process and cell proliferation of Ewing sarcoma. (Right) Epigenetic drug AS-8351 suppresses the function of KDM5B and elevates H3K4me3 level at the promoter region of *FBXW7* gene, then up-regulates FBXW7 and enhances CCNE1 degradation, resulting in cell cycle arrest and tumor growth inhibition.

In conclusion, our study suggests that upregulation of KDM5B plays an important role in the process of cell proliferation in Ewing sarcoma. Specifically, KDM5B inhibits the transcription of *FBXW7* gene by reducing H3K4me3 level at the promoter region, then decreases the FBXW7-mediated degradation of CCNE1, thereby promoting cell cycle progression of EwS cells, which ultimately maintains their malignant phenotypes. The mechanism of KDM5B in Ewing sarcoma is depicted in Fig. 7. Our study further demonstrates the anti-tumor effect of KDM5B inhibitor AS-8351 in Ewing sarcoma. Therefore, targeting KDM5B could be a potential therapy to improve the treatment of Ewing sarcoma. Though further studies are yet warranted, our results suggest the possible usage of epigenetic drugs as an attractive strategy for Ewing sarcoma treatment.

## Supplementary information

Supplementary Figure legends

Supplementary Figure S1

Supplementary Figure S2

Supplementary Table S1

Supplementary Table S2

Supplementary Table S3

Supplementary Table S4

## Data Availability

All data needed to evaluate the conclusions in the paper are present in the paper and/or the Supplementary materials.

## References

[1] Grunewald TGP, Cidre-Aranaz F, Surdez D, Tomazou EM, de Alava E, Kovar H (2018). Ewing sarcoma. Nat Rev Dis Prim.

[2] Riggi N, Suva ML, Stamenkovic I (2021). Ewing’s Sarcoma. N Engl J Med.

[3] Womer RB, West DC, Krailo MD, Dickman PS, Pawel BR, Grier HE (2012). Randomized controlled trial of interval-compressed chemotherapy for the treatment of localized Ewing sarcoma: a report from the Children’s Oncology Group. J Clin Oncol.

[4] Ladenstein R, Potschger U, Le Deley MC, Whelan J, Paulussen M, Oberlin O (2010). Primary disseminated multifocal Ewing sarcoma: results of the Euro-EWING 99 trial. J Clin Oncol.

[5] Van Mater D, Wagner L (2019). Management of recurrent Ewing sarcoma: challenges and approaches. Onco Targets Ther.

[6] Riggi N, Stamenkovic I (2007). The biology of Ewing sarcoma. Cancer Lett.

[7] Lawlor ER, Thiele CJ (2012). Epigenetic changes in pediatric solid tumors: promising new targets. Clin Cancer Res.

[8] Jones PA, Issa JP, Baylin S (2016). Targeting the cancer epigenome for therapy. Nat Rev Genet.

[9] Riggi N, Knoechel B, Gillespie SM, Rheinbay E, Boulay G, Suvà ML (2014). EWS-FLI1 utilizes divergent chromatin remodeling mechanisms to directly activate or repress enhancer elements in Ewing sarcoma. Cancer Cell.

[10] Tomazou EM, Sheffield NC, Schmidl C, Schuster M, Schönegger A, Datlinger P (2015). Epigenome mapping reveals distinct modes of gene regulation and widespread enhancer reprogramming by the oncogenic fusion protein EWS-FLI1. Cell Rep.

[11] Richter GH, Plehm S, Fasan A, Rossler S, Unland R, Bennani-Baiti IM (2009). EZH2 is a mediator of EWS/FLI1 driven tumor growth and metastasis blocking endothelial and neuro-ectodermal differentiation. Proc Natl Acad Sci USA.

[12] Sankar S, Theisen ER, Bearss J, Mulvihill T, Hoffman LM, Sorna V (2014). Reversible LSD1 inhibition interferes with global EWS/ETS transcriptional activity and impedes Ewing sarcoma tumor growth. Clin Cancer Res.

[13] Cloos PA, Christensen J, Agger K, Helin K (2008). Erasing the methyl mark: histone demethylases at the center of cellular differentiation and disease. Genes Dev.

[14] Johansson C, Tumber A, Che K, Cain P, Nowak R, Gileadi C (2014). The roles of Jumonji-type oxygenases in human disease. Epigenomics..

[15] Højfeldt JW, Agger K, Helin K (2013). Histone lysine demethylases as targets for anticancer therapy. Nat Rev Drug Discov.

[16] Park SY, Park JW, Chun YS (2016). Jumonji histone demethylases as emerging therapeutic targets. Pharm Res.

[17] Wang L, Chang J, Varghese D, Dellinger M, Kumar S, Best AM (2013). A small molecule modulates Jumonji histone demethylase activity and selectively inhibits cancer growth. Nat Commun.

[18] Parrish JK, McCann TS, Sechler M, Sobral LM, Ren W, Jones KL (2018). The Jumonji-domain histone demethylase inhibitor JIB-04 deregulates oncogenic programs and increases DNA damage in Ewing Sarcoma, resulting in impaired cell proliferation and survival, and reduced tumor growth. Oncotarget..

[19] Shi Y, Whetstine JR (2007). Dynamic regulation of histone lysine methylation by demethylases. Mol Cell.

[20] Barrett A, Madsen B, Copier J, Lu PJ, Cooper L, Scibetta AG (2002). PLU-1 nuclear protein, which is upregulated in breast cancer, shows restricted expression in normal human adult tissues: a new cancer/testis antigen?. Int J Cancer.

[21] Schmitz SU, Albert M, Malatesta M, Morey L, Johansen JV, Bak M (2011). Jarid1b targets genes regulating development and is involved in neural differentiation. Embo J.

[22] Han M, Xu W, Cheng P, Jin H, Wang X (2017). Histone demethylase lysine demethylase 5B in development and cancer. Oncotarget..

[23] Zheng YC, Chang J, Wang LC, Ren HM, Pang JR, Liu HM (2019). Lysine demethylase 5B (KDM5B): a potential anti-cancer drug target. Eur J Med Chem.

[24] Cao N, Huang Y, Zheng J, Spencer CI, Zhang Y, Fu JD (2016). Conversion of human fibroblasts into functional cardiomyocytes by small molecules. Science..

[25] Keyomarsi K, Pardee AB (1993). Redundant cyclin overexpression and gene amplification in breast cancer cells. Proc Natl Acad Sci USA.

[26] Müller-Tidow C, Metzger R, Kügler K, Diederichs S, Idos G, Thomas M (2001). Cyclin E is the only cyclin-dependent kinase 2-associated cyclin that predicts metastasis and survival in early stage non-small cell lung cancer. Cancer Res.

[27] Akama Y, Yasui W, Yokozaki H, Kuniyasu H, Kitahara K, Ishikawa T (1995). Frequent amplification of the cyclin E gene in human gastric carcinomas. Jpn J Cancer Res.

[28] Iida H, Towatari M, Tanimoto M, Morishita Y, Kodera Y, Saito H (1997). Overexpression of cyclin E in acute myelogenous leukemia. Blood..

[29] Schraml P, Bucher C, Bissig H, Nocito A, Haas P, Wilber K (2003). Cyclin E overexpression and amplification in human tumours. J Pathol.

[30] Bortner DM, Rosenberg MP (1997). Induction of mammary gland hyperplasia and carcinomas in transgenic mice expressing human cyclin E. Mol Cell Biol.

[31] Koepp DM, Schaefer LK, Ye X, Keyomarsi K, Chu C, Harper JW (2001). Phosphorylation-dependent ubiquitination of cyclin E by the SCFFbw7 ubiquitin ligase. Science..

[32] Minella AC, Welcker M, Clurman BE (2005). Ras activity regulates cyclin E degradation by the Fbw7 pathway. Proc Natl Acad Sci USA.

[33] Siu KT, Rosner MR, Minella AC (2012). An integrated view of cyclin E function and regulation. Cell Cycle.

[34] Yeh CH, Bellon M, Nicot C (2018). FBXW7: a critical tumor suppressor of human cancers. Mol Cancer.

[35] Yumimoto K, Nakayama KI (2020). Recent insight into the role of FBXW7 as a tumor suppressor. Semin Cancer Biol.

[36] Feinberg AP, Koldobskiy MA, Göndör A (2016). Epigenetic modulators, modifiers and mediators in cancer aetiology and progression. Nat Rev Genet.

[37] Lawrence MS, Stojanov P, Polak P, Kryukov GV, Cibulskis K, Sivachenko A (2013). Mutational heterogeneity in cancer and the search for new cancer-associated genes. Nature..

[38] Kidder BL, Hu G, Yu ZX, Liu C, Zhao K (2013). Extended self-renewal and accelerated reprogramming in the absence of Kdm5b. Mol Cell Biol.

[39] Catchpole S, Spencer-Dene B, Hall D, Santangelo S, Rosewell I, Guenatri M (2011). PLU-1/JARID1B/KDM5B is required for embryonic survival and contributes to cell proliferation in the mammary gland and in ER+ breast cancer cells. Int J Oncol.

[40] Albert M, Schmitz SU, Kooistra SM, Malatesta M, Morales Torres C, Rekling JC (2013). The histone demethylase Jarid1b ensures faithful mouse development by protecting developmental genes from aberrant H3K4me3. PLoS Genet.

[41] Xhabija B, Kidder BL (2019). KDM5B is a master regulator of the H3K4-methylome in stem cells, development and cancer. Semin Cancer Biol.

[42] Wang Z, Tang F, Qi G, Yuan S, Zhang G, Tang B (2015). KDM5B is overexpressed in gastric cancer and is required for gastric cancer cell proliferation and metastasis. Am J Cancer Res.

[43] Evan GI, Vousden KH (2001). Proliferation, cell cycle and apoptosis in cancer. Nature..

[44] Suva ML, Riggi N, Bernstein BE (2013). Epigenetic reprogramming in cancer. Science.

[45] Zhang ZG, Zhang HS, Sun HL, Liu HY, Liu MY, Zhou Z (2019). KDM5B promotes breast cancer cell proliferation and migration via AMPK-mediated lipid metabolism reprogramming. Exp Cell Res.

