# Supplementary material for: KDM5B promotes tumorigenesis of Ewing sarcoma via FBXW7/CCNE1 axis
Source: Cell Death Dis. 2022 Apr 15;13(4):354. doi: 10.1038/s41419-022-04800-1 (PMC9012801; doi:10.1038/s41419-022-04800-1)
Supplement: Supplementary file 1 — Supplementary Figure legends [file 41419_2022_4800_MOESM1_ESM.docx]

**Supplementary Figure Legends**

**Supplementary** **Figure S1.** **Bioinformatics analysis.** (A) Volcano plot of GSE17674 showing the differentially expressed genes in Ewing Sarcoma comparing with the normal tissues with a total of 2,998 up-regulated expressed genes and 2,300 down-regulated expressed genes. (B) GO pathway analysis of top 1000 genes significantly related to *KDM5B* in a merged dataset of GSE17674, GSE12102 and GSE34620. (C) KEGG enrichment analysis of top 1000 genes significantly related to *KDM5B* in a merged dataset of GSE17674, GSE12102 and GSE34620. (D) GSEA analysis of genes related to *KDM5B* in a merged dataset of GSE17674, GSE12102 and GSE34620.

**Supplementary** **Figure S2. Unprocessed western blot images from all western blot analyses in the whole study.**
