# Supplementary figures and images for: KDM5B promotes tumorigenesis of Ewing sarcoma via FBXW7/CCNE1 axis
Source: Cell Death Dis. 2022 Apr 15;13(4):354. doi: 10.1038/s41419-022-04800-1 (PMC9012801; doi:10.1038/s41419-022-04800-1)

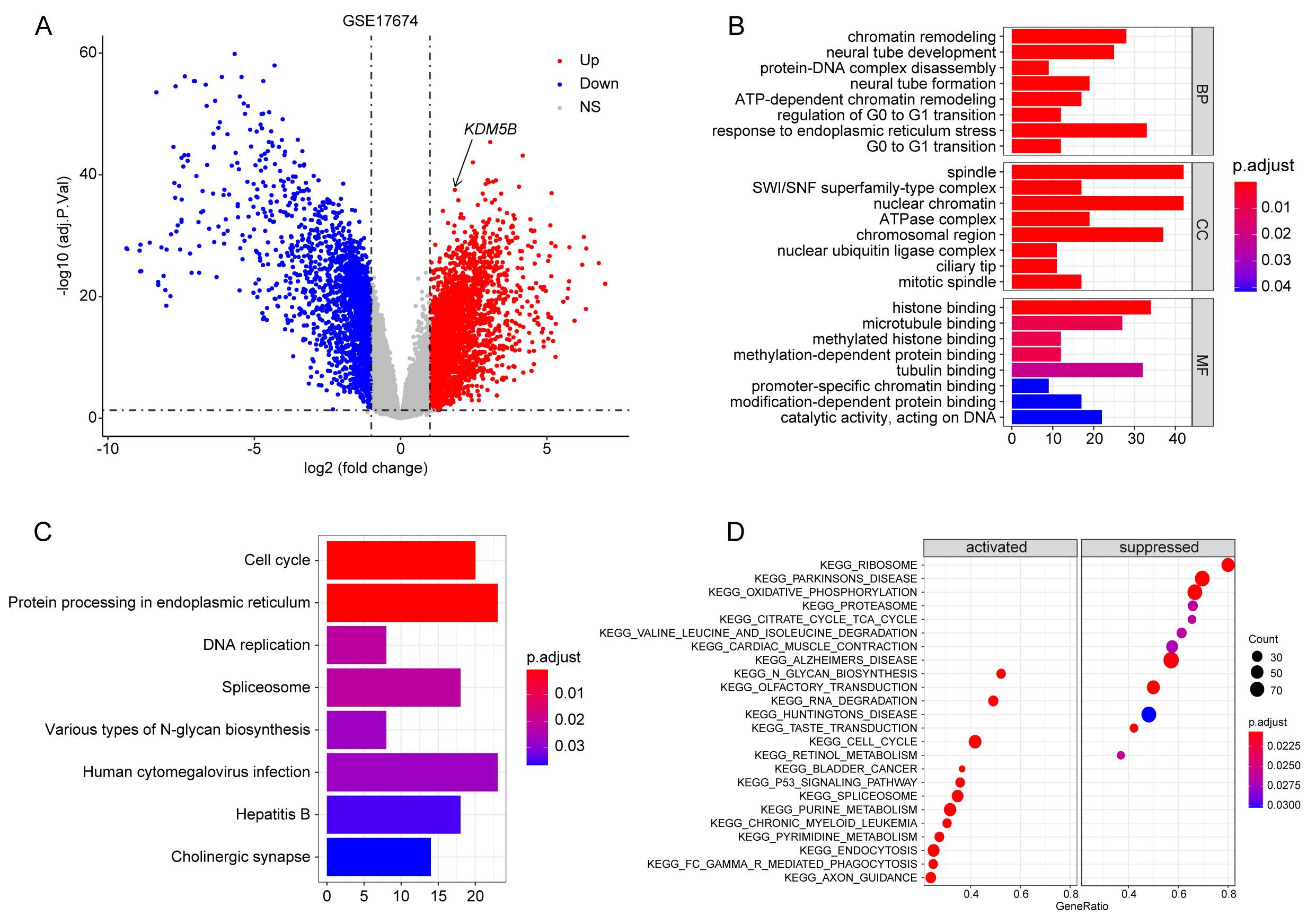

Supplement: Supplementary file 2 — Supplementary Figure S1 [file 41419_2022_4800_MOESM2_ESM.tif]

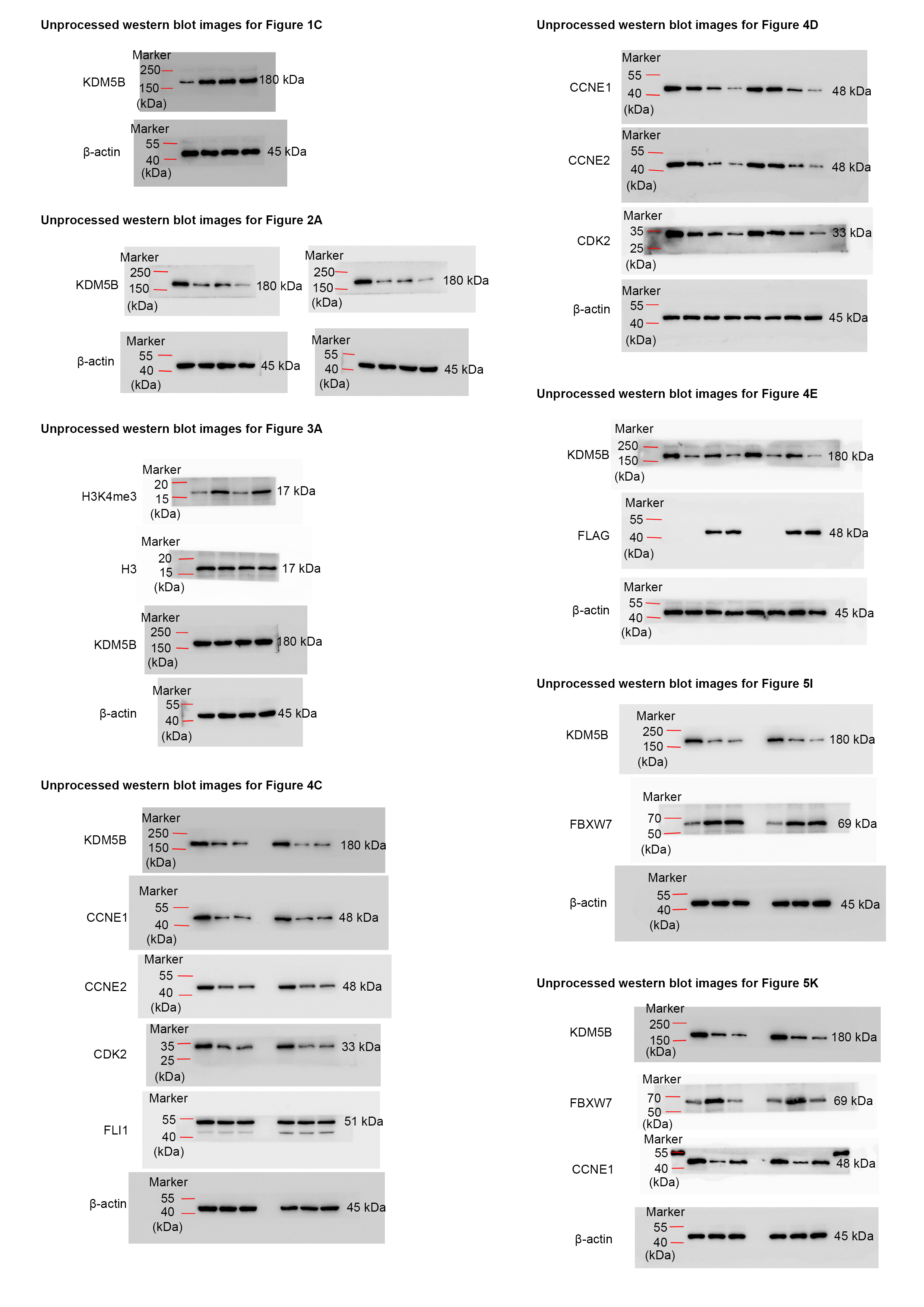

Supplement: Supplementary file 3 — Supplementary Figure S2 [file 41419_2022_4800_MOESM3_ESM.tif]
