# Supplementary material for: KDM5B promotes tumorigenesis of Ewing sarcoma via FBXW7/CCNE1 axis
Source: Cell Death Dis. 2022 Apr 15;13(4):354. doi: 10.1038/s41419-022-04800-1 (PMC9012801; doi:10.1038/s41419-022-04800-1)
Supplement: Supplementary file 4 — Supplementary Table S1 [file 41419_2022_4800_MOESM4_ESM.docx]

Supplementary Table S1 The primers used in study

| Gene | Forward primer (5’-3’) | Reverse primer (5’-3’) |
| --- | --- | --- |
| *KDM5B* | TGTCCGTAAATTGGGAGTG | GATGCAGGCAAACAAGAAG |
| *CCNE1* | TTGTGTCCTGGCTGAATGTATA | AAGGAAATTCAAGGCAGTCAAC |
| *FBXW7* | GTCTGAGAACATTAGTGGGACA | ACTTTGAGTGTCCGATCTGTAG |
| *GAPHD* | GGAGCGAGATCCCTCCAAAAT | GGCTGTTGTCATACTTCTCATGG |
| *FBXW7*  ChIP-qPCR | TTGCCACTGAAACTTGAGCC | TCTCCACAGAACAGGCAAGT |
