# Supplementary material for: KDM5B promotes tumorigenesis of Ewing sarcoma via FBXW7/CCNE1 axis
Source: Cell Death Dis. 2022 Apr 15;13(4):354. doi: 10.1038/s41419-022-04800-1 (PMC9012801; doi:10.1038/s41419-022-04800-1)
Supplement: Supplementary file 5 — Supplementary Table S2 [file 41419_2022_4800_MOESM5_ESM.docx]

Supplementary Table S2 Characteristics of 46 patients with Ewing sarcoma at our center

| Characteristics | Numbers (%) |
| --- | --- |
| Age when diagnosis |  |
| median (years) | 12 |
| range (years) | 1-17 |
| Sex |  |
| Male | 31 (67.4) |
| Female | 15 (32.6) |
| EWSR1-FLI1 fusion |  |
| Positive | 16 (34.8) |
| Negative | 4 (8.7) |
| Unknown | 26 (56.5) |
| Primary site |  |
| Head and neck | 5 (10.9) |
| Chest | 10 (21.7) |
| Abdomen and pelvis | 12 (26.1) |
| Brain and spinal cord | 8 (17.4) |
| Limbs | 11 (23.9) |
| Origin site |  |
| Extraskeletal location | 44 (95.7) |
| Skeletal location | 2 (4.3) |
| Maximum diameter of tumor |  |
| ≤ 50mm | 14 (30.4) |
| > 50mm | 32 (69.6) |
| Clinical stage |  |
| Limited stage | 40 (87.0) |
| Extensive stage | 6 (13.0) |
| Surgery |  |
| Yes | 41 (89.1) |
| No | 5 (10.9) |
| Chemotherapy |  |
| Yes | 41 (89.1) |
| No | 5 (10.9) |
| Chemotherapy courses |  |
| ≥ 6 courses | 36 (78.3) |
| < 6 courses | 10 (21.7) |
| Radiotherapy |  |
| Yes | 23 (50.0) |
| No | 23 (50.0) |
| Therapeutic Modalities |  |
| Comprehensive therapy | 38 (82.6) |
| Chemotherapy + surgery + radiotherapy | 21 (45.7) |
| Chemotherapy + surgery | 15 (32.6) |
| Chemotherapy + radiotherapy | 2 (4.3) |
| Monotherapy | 8 (17.4) |
| Chemotherapy | 3 (6.5) |
| Surgery | 5 (10.9) |

Values are presented as numbers of patients with percentage in parentheses.
