# Supplementary material for: KDM5B promotes tumorigenesis of Ewing sarcoma via FBXW7/CCNE1 axis
Source: Cell Death Dis. 2022 Apr 15;13(4):354. doi: 10.1038/s41419-022-04800-1 (PMC9012801; doi:10.1038/s41419-022-04800-1)
Supplement: Supplementary file 6 — Supplementary Table S3 [file 41419_2022_4800_MOESM6_ESM.docx]

Supplementary Table S3 Comparison of clinical characteristics between negative and positive expression of KDM5B

| Characteristics | Expression of KDM5B | | *P* value |
| --- | --- | --- | --- |
|  | Negative (n=26) | Positive (n=20) |  |
| Sex |  |  | 0.762 |
| Male | 18 (69.2) | 13 (65.0) |  |
| Female | 8 (30.8) | 7 (35.0) |  |
| Age |  |  | 0.393 |
| ≤ 12 years old | 15 (57.7) | 9 (45.0) |  |
| > 12 years old | 11 (42.3) | 11 (55.0) |  |
| Maximum diameter of tumor |  |  | 0.955 |
| ≤ 50mm | 8 (30.8) | 6 (30.0) |  |
| > 50mm | 18 (69.2) | 14 (70.0) |  |
| Primary site |  |  | 0.271 |
| Head and neck | 3 (11.5) | 2 (10.0) |  |
| Chest | 6 (23.1) | 4 (20.0) |  |
| Abdomen and pelvis | 5 (19.2) | 7 (35.0) |  |
| Brain and spinal cord | 3 (11.5) | 5 (25.0) |  |
| Limbs | 9 (34.6) | 2 (10.0) |  |
| Origin site |  |  | 1.000 |
| Extraskeletal location | 25 (96.2) | 19 (95.0) |  |
| Skeletal location | 1 (3.8) | 1 (5.0) |  |
| Clinical stage |  |  | 0.212 |
| Limited stage | 21 (80.8) | 19 (95.0) |  |
| Extensive stage | 5 (19.2) | 1 (5.0) |  |
| Surgery |  |  | 1.000 |
| Yes | 23 (88.5) | 18 (90.0) |  |
| No | 3 (11.5) | 2 (10.0) |  |
| Chemotherapy |  |  | 0.369 |
| Yes | 22 (84.6) | 19 (95.0) |  |
| No | 4 (15.4) | 1 (5.0) |  |
| Chemotherapy courses |  |  | 1.000 |
| ≥ 6 courses | 20 (76.9) | 16 (80.0) |  |
| < 6 courses | 6 (23.1) | 4 (20.0) |  |
| Radiotherapy |  |  | 0.234 |
| Yes | 11 (42.3) | 12 (60.0) |  |
| No | 15 (57.7) | 8 (40.0) |  |
| Therapeutic Modalities |  |  | 0.113 |
| Comprehensive therapy | 19 (73.1) | 19 (95.0) |  |
| Monotherapy | 7 (26.9) | 1 (5.0) |  |

Values are presented as numbers of patients with percentage in parentheses. *P* values were performed using the Chi-square test.
