# Supplementary material for: KDM5B promotes tumorigenesis of Ewing sarcoma via FBXW7/CCNE1 axis
Source: Cell Death Dis. 2022 Apr 15;13(4):354. doi: 10.1038/s41419-022-04800-1 (PMC9012801; doi:10.1038/s41419-022-04800-1)
Supplement: Supplementary file 7 — Supplementary Table S4 [file 41419_2022_4800_MOESM7_ESM.docx]

Supplementary Table S4 Comparison of clinical characteristics between low and high expression of FBXW7

| Characteristics | Expression of FBXW7 | | *P* value |
| --- | --- | --- | --- |
|  | Low (n=21) | High (n=25) |  |
| Sex |  |  | 0.243 |
| Male | 16 (76.2) | 15 (60.0) |  |
| Female | 5 (23.8) | 10 (40.0) |  |
| Age |  |  | 0.152 |
| ≤ 12 years old | 9 (42.9) | 16 (64.0) |  |
| > 12 years old | 12 (57.1) | 9 (36.0) |  |
| Maximum diameter of tumor |  |  | 0.695 |
| ≤ 50mm | 7 (33.3) | 7 (28.0) |  |
| > 50mm | 14 (66.7) | 18 (72.0) |  |
| Primary site |  |  | 0.973 |
| Head and neck | 2 (9.6) | 3 (12.0) |  |
| Chest | 5 (23.8) | 5 (20.0) |  |
| Abdomen and pelvis | 6 (28.6) | 6 (24.0) |  |
| Brain and spinal cord | 4 (19.0) | 4 (16.0) |  |
| Limbs | 4 (19.0) | 7 (28.0) |  |
| Origin site |  |  | 1.000 |
| Extraskeletal location | 20 (95.2) | 24 (96.0) |  |
| Skeletal location | 1 (4.8) | 1 (4.0) |  |
| Clinical stage |  |  | 1.000 |
| Limited stage | 18 (85.7) | 22 (88.0) |  |
| Extensive stage | 3 (14.3) | 3 (12.0) |  |
| Surgery |  |  | 1.000 |
| Yes | 19 (90.5) | 22 (88.0) |  |
| No | 2 (9.5) | 3 (12.0) |  |
| Chemotherapy |  |  | 0.163 |
| Yes | 17 (81.0) | 24 (96.0) |  |
| No | 4 (19.0) | 1 (4.0) |  |
| Chemotherapy courses |  |  | 0.035 |
| ≥ 6 courses | 13 (61.9) | 23 (92.0) |  |
| < 6 courses | 8 (38.1) | 2 (8.0) |  |
| Radiotherapy |  |  | 0.767 |
| Yes | 10 (47.6) | 13 (52.0) |  |
| No | 11 (52.4) | 12 (48.0) |  |
| Therapeutic Modalities |  |  | 0.439 |
| Comprehensive therapy | 16 (76.2) | 22 (88.0) |  |
| Monotherapy | 5 (23.8) | 3 (12.0) |  |

Values are presented as numbers of patients with percentage in parentheses. *P* values were performed using the Chi-square test.
